# Visceral Adiposity as a Significant Predictor of Sunitinib-Induced Dose-Limiting Toxicities and Survival in Patients with Metastatic Clear Cell Renal Cell Carcinoma

**DOI:** 10.3390/cancers12123602

**Published:** 2020-12-02

**Authors:** Jee Soo Park, Kyo Chul Koo, Doo Yong Chung, Sun Il Kim, Jeongho Kim, Cheol Kyu Oh, Tae Nam Kim, Sung Ku Kang, Jae Won Park, Young Eun Yoon, Sung Yul Park, Koon Ho Rha, Won Sik Ham

**Affiliations:** 1 Department of Urology and Urological Science Institute, Yonsei University College of Medicine, Seoul 03722, Korea; sampark@yuhs.ac (J.S.P.); khrha@yuhs.ac (K.H.R.); 2 Department of Urology, Gangnam Severance Hospital, Yonsei University College of Medicine, Seoul 06273, Korea; gckoo@yuhs.ac; 3 Department of Urology, Inha University College of Medicine, Incheon 22212, Korea; dychung@inha.ac.kr; 4 Department of Urology, Ajou University School of Medicine, Suwon 16499, Korea; sikimuro@ajou.ac.kr; 5 Department of Urology, Inje University Haeundae Paik Hospital, Inje University College of Medicine, Pusan 47392, Korea; h00532@paik.ac.kr (J.K.); h00118@paik.ac.kr (C.K.O.); 6 Department of Urology, Medical Research Institute, Pusan National University Hospital, Pusan 50612, Korea; bigman1995@pusan.ac.kr; 7 Department of Urology, National Health Insurance Service Ilsan Hospital, Goyang 10444, Korea; urokang@nhimc.or.kr (S.K.K.); epria@nhimc.or.kr (J.W.P.); 8 Department of Urology, Hanyang University College of Medicine, Seoul 04763, Korea; urologistyoon@hanyang.ac.kr (Y.E.Y.); syparkuro@hanyang.ac.kr (S.Y.P.)

**Keywords:** dose-limiting toxicity, obesity, renal cell carcinoma, sunitinib, visceral adiposity

## Abstract

**Simple Summary:**

Although sunitinib is a standard first-line treatment for metastatic renal cell carcinoma, little is known about the predictive factors of sunitinib-induced dose-limiting toxicity in Asian populations. Thus, the aim of this study was to determine whether body composition features significantly associated with sunitinib toxicity in Western populations might predict early dose-limiting toxicity in Korean patients with metastatic renal cell carcinoma. The administration of sunitinib at a fixed dose has the advantage of simplicity but does not consider inter-individual variations, resulting in a high drug toxicity. As high-resolution computed tomography images of patients with cancer are readily available using the body composition, mainly visceral adipose tissue, individualized sunitinib dosage could be feasible in real-world clinical practice, according to this study. Using the appropriate sunitinib dosage based on body composition, visceral adipose tissue index, would reduce sunitinib toxicity.

**Abstract:**

Sunitinib is a first-line treatment for metastatic renal cell carcinoma (mRCC). Little is known about the predictive factors of sunitinib-induced dose-limiting toxicity (DLT) in Asian populations. We investigated whether body composition predicts sunitinib-induced DLT. We retrospectively reviewed sunitinib-treated Korean patients with clear cell mRCC from eight institutions. Body composition was measured using computed tomography. DLT was defined as any adverse event leading to dose reduction or treatment discontinuation. Univariate analysis was used to compare body composition indices, and logistic regression analyses were performed for factors predicting early DLT. Overall, 111/311 (32.5%) of patients experienced DLT. Significant differences were observed in the subcutaneous adipose tissue index (SATI; *p* = 0.001) and visceral adipose tissue index (VATI; *p* < 0.001) between patients with and without DLT. Multivariate analyses revealed that VATI (odds ratio: 1.013; *p* = 0.029) was significantly associated with early DLT. Additionally, 20% of patients who had a body mass index (BMI) greater than 23 kg/m^2^ and a low VATI experienced DLT, whereas 34.3% of the remaining groups had DLT (*p* = 0.034). Significant differences were observed for median progression-free survival (13.0 vs. 26.0 months, respectively; *p* = 0.006) between patients with low and high VATI. Visceral adiposity was a significant predictor of sunitinib-associated DLT and survival. Patients with a low VATI and a BMI greater than 23 kg/m^2^ experienced lower DLTs.

## 1. Introduction

Renal cell carcinoma (RCC) is the most common kidney cancer, and clear cell RCC (cc-RCC) is the most prevalent type worldwide [1]. The incidence and mortality rates are rising 2–3% per decade; around 30% of patients are diagnosed in the metastatic stage, with <10% surviving more than 5 years [2,3]. For advanced metastatic RCC (mRCC), sunitinib (Sutent; Pfizer), a multikinase inhibitor with antiangiogenic properties, is a standard first-line treatment option [4,5].

Common toxic effects of sunitinib are diarrhea, hand-foot syndrome, fatigue, and hypertension, which limit the full dose of sunitinib and account for dose reduction and treatment termination in 32% and 8% of patients, respectively [4]. Little is known about the factors that predict sunitinib toxicity in RCC [6]. There is substantial inter-individual variability in sunitinib pharmacokinetics, which is partly explained by the varying cytochrome P450 (CYP450) expression levels and CYP3A5 polymorphisms [7,8]. This might identify some patients prone to toxicity-related dose reductions that cannot be routinely assessed in the clinic [6,9].

Body composition, including skeletal muscle (SM), adipose tissue, and fat-free mass, is a prognostic factor in cancer and is reported to be superior to conventional measures of body size, such as weight, body mass index (BMI), and body surface area (BSA), for drug dosage prediction [6,10]. Patients with mRCC have diverse BMIs (15.2–38.1 kg/m^2^), resulting in differences in drug plasma concentration and metabolism, leading to different toxicity rates. Nevertheless, sunitinib is administered at a fixed dosage of 50 mg daily for 28 days, followed by a 14-day break, during which body composition is not accounted for [6].

Previous studies have demonstrated how body composition relates to sunitinib toxicity in Western populations [6,9]; however, only one study has been performed in Asian populations, which is inadequate to analyze all body composition features. Therefore, we determined whether body composition features significantly associated with sunitinib toxicity in Western populations might predict early dose-limiting toxicity (DLT) in Korean patients with mRCC.

## 2. Results

### 2.1. Patient Characteristics

We identified 311 patients with cc-mRCC that received sunitinib as a first-line therapy. The baseline characteristics of these patients are presented in Table 1. The median BMI was 23.5 kg/m^2^, and 3.5% of patients were classified as underweight. Collectively, 76.8% of tumors were classified as high-grade.

### 2.2. Sunitinib Toxicity

During the first cycle of treatment, 101 patients (32.5%) experienced a DLT. Except in 38 cases, patients experienced multiple grade 2 to 4 adverse events (AEs). The most frequent AEs were fatigue (17.2%), hand-foot syndrome (17.0%), and thrombocytopenia (8.8%). Other AEs, including grade 1 and 2 gastrointestinal AEs and grade 2 and 3 hypertension, occurred frequently.

For all patients with DLTs, sunitinib was discontinued and resumed at a lower dose (37.5 mg). Seven cases of permanent discontinuation were reported, owing to DLT during the first cycle of treatment. Dose reduction in 12 patients did not sufficiently alleviate symptoms and their sunitinib was discontinued.

The comparison of anthropometric parameters between patients with or without early DLT is summarized in Table 2. Those patients with a DLT had a significantly higher subcutaneous adipose tissue (SAT) index (SATI; *p* = 0.001) and visceral adipose tissue (VAT) index (VATI; *p* < 0.001) than patients without a DLT. A multivariate logistic regression analysis of indices that were significantly associated with early DLT in univariate analysis revealed that only a patient’s VATI (odds ratio (OR): 1.013, 95% confidence interval (CI): 1.001–1.025, *p* = 0.029) was significantly associated with early DLT, whereas their SATI (OR: 1.009, 95% CI: 0.998–1.020, *p* = 0.119) was not. Furthermore, parameters associated with SM were not associated with early DLT, even in logistic regression analysis (skeletal muscle index (SMI) (OR: 0.986, 95% CI: 0.959–1.015, *p* = 0.350); SM (OR: 0.995, 95% CI: 0.987–1.003, *p* = 0.207)).

Compared to patients with a low VATI, those with a high VATI experienced more DLTs (23.9% vs. 41.0%, respectively; *p* = 0.001) and AEs (*p* < 0.001) (Table 3).

**Figure 1:**
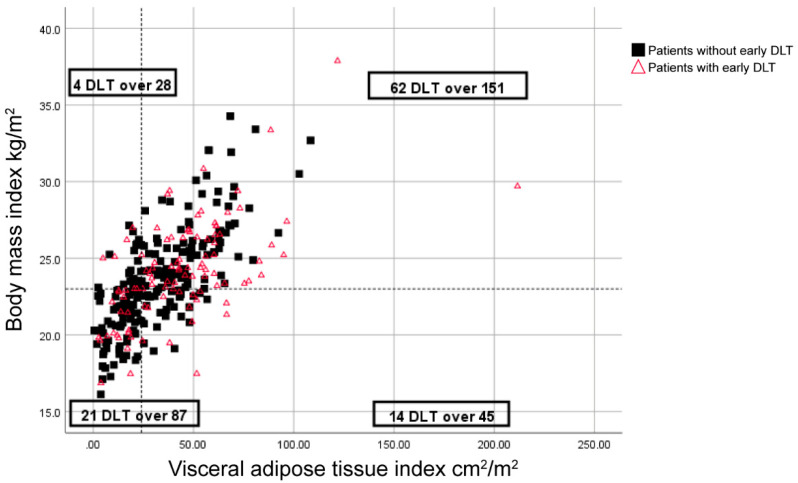
Distribution of body mass index (BMI), visceral adipose tissue index (VATI), and early dose-limiting toxicity (DLT) for sunitinib-treated patients. Symbols represent individual patients. The vertical dashed-line indicates the cut-off point for low VATI (<24 cm^2^/m^2^), and the horizontal dashed line indicates a BMI of 23 kg/m^2^.

Moreover, DLT was reported in 20% of patients with a BMI greater than 23 kg/m^2^ and a low VATI (<24 cm^2^/m^2^), whereas the remaining 34.3% of patients had DLTs (*p* = 0.034) (Figure 1).

### 2.3. Survival Analysis

**Figure 2:**
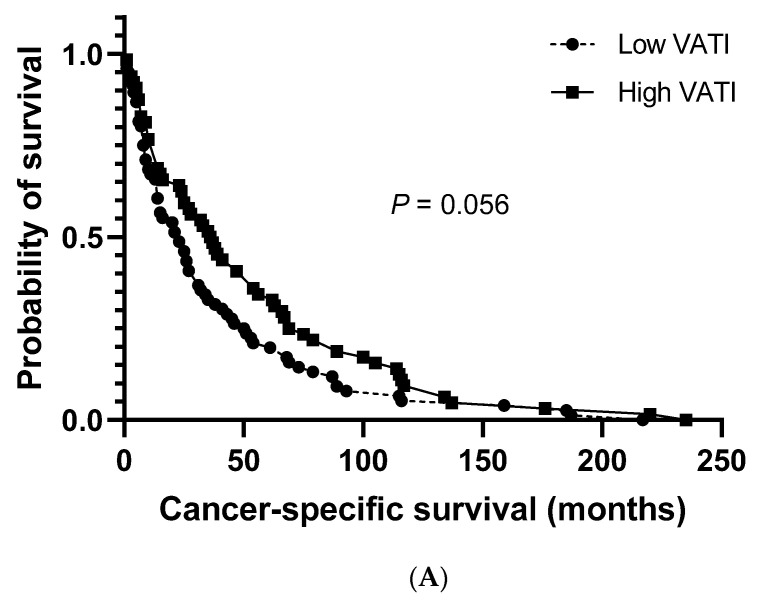

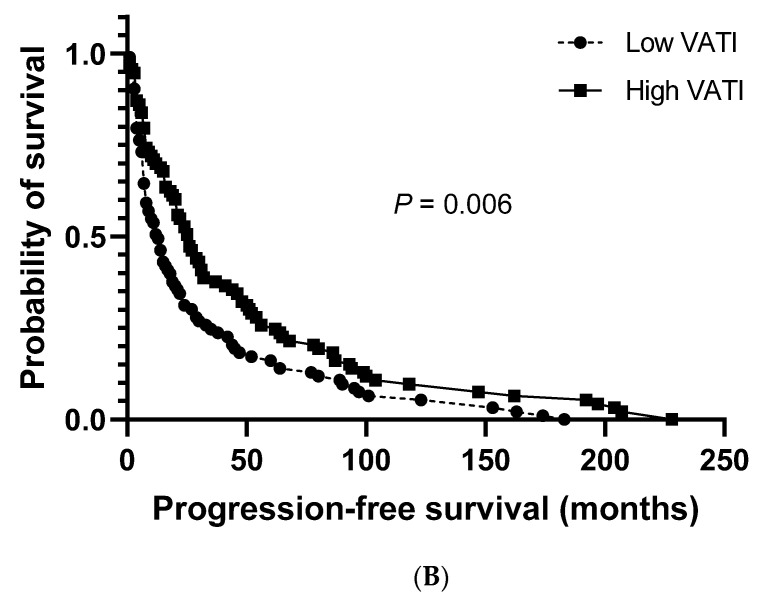
Overall survival. (**A**) Cancer-specific survival in patients with high/low visceral adipose tissue index (VATI). (**B**) Progression-free survival in patients with high/low VATI.

The median cancer-specific survival (CSS) and progression-free survival (PFS) for the study population were 27.0 months (95% CI: 20.8–33.2) and 19.0 months (95% CI: 14.3–23.7), respectively. No significant differences were observed between patients with a low and high VATI regarding the median CSS (23.0 months (95% CI: 13.5–32.5) vs. 36.0 months (95% CI: 23.3–48.7), respectively; Figure 2A). However, a significantly shorter median PFS was observed in patients with a low VATI than in patients with a high VATI (13.0 months (95% CI: 8.6–17.4) vs. 26.0 months (95% CI: 19.1–32.9), respectively; *p* = 0.006; Figure 2B).

## 3. Discussion

This is the first large, nationwide, multi-institutional study to identify the relationship between the VATI and early toxicity in Korean sunitinib-treated patients with cc-mRCC. Unlike other groups that have shown an association between sarcopenia and sunitinib-induced DLT, we observed that patients with a high VATI experienced significantly more DLTs than those with a low VATI during the first cycle.

Four studies have evaluated the toxicity of antiangiogenic agents (sorafenib [10] and sunitinib [6,9,11]) in patients with mRCC. A meta-analysis of these studies demonstrated that DLT is more frequent in patients with a low SMI, with or without a BMI less than 25 kg/m^2^, than in patients with a high SMI or a BMI greater than 25 kg/m^2^ [12]. However, most of these studies were performed on Caucasians [6,9,10], and only one study focused on a Japanese population [11]. Despite being the only study performed on an Asian population, Ishihara et al. did not evaluate if adipose tissues affected the safety and tolerability of sunitinib. In the two studies that have evaluated the effect of body composition on DLT in sunitinib-treated patients, the study populations exhibited a wide range of BMI and muscularity—i.e., characteristics of “westernized” societies. These studies observed that 34.5% [6] and 21.3% [9] of patients were obese, whereas 4.2% of our patients were obese.

The cut-off values for BMI and sarcopenia have been based on Western populations and should be adjusted for Asian populations. While the associations among BMI, body composition, and health risks are different between these populations, the universal BMI criteria developed by the World Health Organization (WHO) might be unsuitable for Asian populations [13]. Anuurad et al. re-categorized BMI criteria for Asian populations as follows: underweight (<18.5 kg/m^2^), normal (18.5–22.9 kg/m^2^), overweight (23–24.9 kg/m^2^), and obese (>25 kg/m^2^) [14]. Therefore, we used the BMI 23 kg/m^2^ cut-off to classify a patient as overweight, instead of the metrics used in Western studies [9,10].

Our study showed that the 23 kg/m^2^-cut-off was more appropriate. When using the 23 kg/m^2^ cut-off, no DLT was reported in 20% of patients, whereas, when using the 25 kg/m^2^ cut-off, no DLT was reported in 27.3% of patients. The difference in the probability of a DLT between patients who simultaneously had a BMI higher than the cut-off point and a low VATI compared with the remaining patients was significant (*p* = 0.034) when using the 23 kg/m^2^ cut-off, whereas the 25 kg/m^2^ cut-off showed no significant difference. Therefore, we suggest using these parameters for Asian populations. While comparing our results with those of Ishihara et al. would help to establish a standard cut-off for Asian populations, the Ishihara et al. study did not report the values for adipose tissue.

Although there were no significant associations between SM parameters and DLT in our study, previous studies have reported that SM, especially sarcopenia, is associated with DLT; however, these studies used different definitions of sarcopenia [6,9,10,11] or did not apply the international definition, which might have resulted in bias. An international, standard definition for sarcopenia needs to be established [15], and our results can contribute to international standard definitions by considering the body composition of Asian populations.

In our study, there was no significant association between SM, or SMI and DLT, possibly due to different body compositions. Among patients with a low VATI, only overweight or obese patients experienced fewer DLT, whereas normal or underweight patients experienced more DLT. Similarly, studies have shown the link between a low BMI and chemotherapy-related toxicity [16,17]. However, there was no significant difference (*p* = 0.697) between BMI and the probability of DLT in patients with a low VATI.

We observed inconsistencies when BMI was used as an obesity marker. BMI encompasses several important parameters, including sarcopenia, VAT, and SAT, which explains some observations. A high BMI increases the risk of RCC [18]. The relationship between BMI and RCC prognosis remains controversial, as some studies report a diverse range of associations [19]. To understand how obesity affects cancer progression and drug-related toxicity, combining the VAT, SAT, and SM parameters is needed, depending on the cancer type and the patient’s status.

In our study, VATI was a significant predictor of early DLT. Sunitinib is predominantly metabolized via the liver-based CYP450 enzyme, CYP3A4 [20]. VAT is a strong, independent risk factor for elevated liver transaminase levels and fatty liver [21], which are associated with decreased CYP3A4 expression and activity. Therefore, a rise in patients with a DLT and high VAT content is due to the decreased metabolism of sunitinib via CYP3A4 [22,23]. Subsequently, these patients would have high sunitinib concentrations, which explains the association between more patients with a DLT and a high VATI. Future studies are needed to characterize the association between sunitinib pharmacokinetics and VAT and SM [12].

Five studies have examined VAT effects in patients with localized and advanced disease. Low VAT content was associated with poor prognosis [24,25,26]. Another reported no association with overall mortality [27] and, in the other, the lowest and highest quartiles of VAT percentage were associated with a higher risk of recurrence [19]. VAT associates with the largest endocrine organ that produces hormones and cytokines related to cancer progression [28]. VAT releases adipokines, including adiponectin, that have anti-inflammatory and angiogenesis-inhibiting effects [29]. Low adiponectin levels correspond with large tumor size and metastasis in RCC, and its levels inversely correlate with Fuhrman nuclear grades [30]. Adiponectin levels relate more to VAT [31], which supports the association between a high VATI and prolonged PFS.

Our study had several limitations. Owing to the retrospective nature of our analysis, some results might be biased, especially as we only evaluated Korean patients. Moreover, we have not documented and compared medications that might have influenced the pharmacokinetics of sunitinib or DLT. Studies to validate this finding in other Asian populations are needed.

## 4. Materials and Methods

### 4.1. Patients and Ethics

This retrospective review of a multi-institutional prospective cohort study was approved by the institutional review boards and human research ethics committees of each participating institution (project no: 4-2020-00528). Among participants in this prospective cohort of patients treated with cc-mRCC targeted therapy from eight tertiary medical centers in Korea between November 2005 and November 2019, those treated with first-line sunitinib treatments were evaluated. All the procedures involving human participants were performed in accordance with institutional and national ethical standards and the Declaration of Helsinki. For the original prospective cohort study, all the patients provided informed consent, and the manuscript contains no personal data. The datasets used and/or analyzed in this study are available from the corresponding author upon reasonable request.

### 4.2. Study Design

Adult outpatients with cc-mRCC who received sunitinib at an initial dose of 50 mg per day according to their Eastern Cooperative Oncology Group performance status and comorbidities, at the treating physician’s discretion, were included in this study, as previously described [6,9,10,32]. Toxicity profiles were recorded, analyzed, and grouped according to the Common Terminology Criteria for Adverse Events (CTCAE, v5.0). Toxicity was assessed at each visit every 2 weeks (or before if clinically indicated) during the first cycle, then monthly, until the discontinuing of the drug. One treatment cycle was for 6 weeks (4 weeks on and 2 weeks off).

In the case of CTCAE grade 3 or 4 AEs, sunitinib was discontinued, except for grade 3 hypertension, for which antihypertensives were introduced according to guidelines [33]. Depending on toxicity resolution, continuation with the initial or decreased dosage or discontinuation was determined at the treating physician’s discretion.

A DLT was defined as any AEs leading to a dose reduction or the temporary or permanent discontinuation of treatment. Following the design of a previous study [34], only DLTs occurring during the first cycle of treatment (6 weeks) were examined. Progression was defined as local recurrence or distant metastasis, and data on mortality were collected from medical records in the Cancer Registry Center database at our institution.

### 4.3. Anthropometry and Body Composition

Weights and heights were recorded during clinical visits according to standard methods, and recorded values closest to the patient’s computed tomography (CT) scan date were used for analysis. BMI was calculated (weight (kg)/height^2^ (m^2^)) and WHO categories were used: underweight, BMI < 18.5; normal, BMI between 18.5 and 24.9; overweight, BMI between 25 and 29.9; obese, BMI > 30 [9,10]. BSA was calculated using the Mosteller formula: BSA (m^2^) = ((height (cm) × weight (kg))/3600)^1/2^ [9,10,35].

CT, for accurate body composition measurements [36,37], was used to assess regional adipose (visceral and subcutaneous) and muscle tissues. The muscle and adipose tissue values were reported as total muscle and visceral and subcutaneous fat cross-sectional areas at the third lumbar vertebra (L3) (cm^2^) [6,9,10]. The indices used were divided by the patient’s height squared (cm^2^/m^2^) to normalize the values [38].

### 4.4. Image Analysis

**Figure 3:**
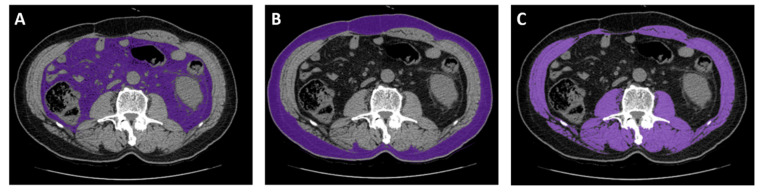
Representative computed tomography measurements of tissues using analysis software. (**A**) Visceral adipose tissue. (**B**) Subcutaneous adipose tissue. (**C**) Skeletal muscle.

CT images captured near the start of the sunitinib treatment (within 30 days) were considered. CT scans were excluded if any interventions, such as surgery, that altered body composition occurred between the CT scan and starting sunitinib treatment. CT images were analyzed using an Aquarius iNtuition Viewer, v.4.4.12 (TeraRecon). Different body compositions were evaluated using predefined Hounsfield unit (HU) thresholds. SM was evaluated using thresholds of −29–150 HU [6,9]. For SAT, −190 to −30 HU was used, and for VAT, −150 to −50 HU was used (Figure 3) [6].

### 4.5. Statistical Analyses

Data are presented as the mean ± standard deviation or median (interquartile range) for continuous variables and a percentage for categorical variables. A high VATI was higher than the median VATI value. For univariate analysis, a Wilcoxon test or Student’s *t*-test was used to compare continuous variables, and a chi-square test or Fisher’s exact test was used to compare categorical variables. Logistic regression analyses were performed for factors predicting early DLTs, including factors that predicted the occurrence of DLTs at a *p* < 0.05 upon univariate analysis and ORs with 95% CIs. PFS and CSS were measured from the first sunitinib administration to the date of disease progression or death. Kaplan–Meier estimates of the distribution of times from the baseline to outcome were computed, and groups were compared using the log-rank test. SPSS software v.23.0 (IBM Corp., Armonk, NY, USA) and GraphPad Prism 8.0 software (GraphPad Software, Inc., La Jolla, CA, USA) were used for all statistical analyses. All the statistical tests were two-tailed, and *p*-values less than 0.05 were statistically significant.

## 5. Conclusions

In this study, VATI was a significant predictor of sunitinib-associated DLT and survival in patients with mRCC; those with a low VATI and a BMI greater than23 kg/m^2^ experienced significantly fewer DLTs during the first cycle of treatment. Future studies will focus on using sunitinib doses based on body composition, mainly VATI, to reduce DLT.

## Figures and Tables

**Table 1:** Baseline characteristics of the patients treated with sunitinib.

Characteristics	Male (*n* = 251)	Female (*n* = 60)	Total (*n* = 311)	*p*
Age, median (range) * (y)	62.0 (56.0–71.0)	64.0 (59.0–73.8)	63.0 (57.0–72.0)	0.134
Weight, median (range) * (kg)	69.0 (61.8–75.7)	56.5 (50.6–60.8)	66.8 (58.0–74.7)	<0.001
BMI, median (range) * (kg/m^2^)	23.7 (21.8–25.8)	22.9 (21.6–25.2)	23.5 (21.8–25.7)	0.260
Underweight (BMI < 18.5)	7 (2.8%)	4(6.7%)	11 (3.5%)	0.112
Normal weight (18.5 ≤ BMI ≤ 24.9)	158 (62.9%)	41 (68.3%)	199 (64.0%)	
Overweight (25 ≤ BMI ≤ 29.9)	77 (30.7%)	11 (18.3%)	88 (28.3%)	
Obese (30 ≤ BMI)	9 (3.6%)	4 (6.7%)	13 (4.2%)	
ECOG PS, *n* (%)				
0–1	239 (95.2%)	56 (93.3%)	295 (94.9%)	0.522
≥2	12 (4.8%)	4 (6.7%)	16 (5.1%)	
Number of metastatic sites, *n* (%)				
1	106 (42.2%)	23 (38.3%)	129 (41.5%)	0.589
2	78 (31.1%)	24 (40.0%)	102 (32.8%)	
3	49 (19.5%)	11 (18.3%)	60 (19.3%)	
≥4	18 (7.2%)	2 (3.3%)	20 (6.4%)	
Metastatic sites, *n* (%)				
Lung	194 (40.6%)	39 (34.5%)	233 (39.4%)	0.800
Liver	44 (11.5%)	11 (48.7%)	55 (9.3%)	
Bone	70 (14.6%)	19 (16.8%)	89 (15.1%)	
Brain	15 (3.1%)	3 (2.7%)	18 (3.0%)	
Others	155 (32.4%)	41 (36.3%)	196 (33.2%)	
Early DLT, *n* (%)				
Present	73 (29.1%)	28 (46.7%)	101 (32.5%)	0.009
Absent	178 (70.9%)	32 (53.3%)	210 (67.5%)	
L3 Area, median (range) * (cm^2^)				
SAT	98.5 (71.6–129.7)	150.2 (123.4–177.6)	110.9 (75.5–145.9)	<0.001
VAT	105.4 (56.0–150.7)	69.8 (43.7–115.2)	95.4 (51.1–146.4)	0.009
SM	145.1 (130.7–160.9)	98.2 (90.0–106.2)	139.6 (114.2–155.3)	<0.001
L3 Index, median (range) * (cm^2^/m^2^)				
SATI	34.5 (24.1–46.2)	62.5 (48.6–72.0)	38.1 (26.0–55.2)	<0.001
VATI	36.1 (18.6–52.1)	30.0 (17.9–46.5)	34.3 (18.5–51.7)	0.381
SMI	50.5 (45.4–55.1)	40.6 (36.9–43.9)	48.2 (42.2–54.1)	<0.001
No. ISUP grade, *n* (%)				
Low grade (1–2)	52 (20.7%)	20 (33.3%)	72 (23.2%)	0.037
High grade (3–4)	199 (79.3%)	40 (66.7%)	234 (76.8%)	

* Quartile range. Data are presented as medians (interquartile ranges) for continuous variables and as percentages for categorical variables. *p*-values are from the chi-square test or Fisher’s exact test when categorical and Wilcoxon’s test when continuous. BMI: body mass index; DLT: dose-limiting toxicity; ECOG PS: Eastern Cooperative Oncology Group criteria performance status; SAT: subcutaneous adipose tissue; SATI: subcutaneous adipose tissue index; SM: skeletal muscle; SMI: skeletal muscle index; VAT: visceral adipose tissue; VATI: visceral adipose tissue index.

**Table 2:** Comparison of anthropometric measurements and demographic characteristics of patients with or without early DLT.

	Patients with Early DLT	Patients without Early DLT	*p*
	(*n* = 101)	(*n* = 210)	
Age (y)	65.2 (11.8)	62.8 (9.7)	NS
Weight (kg)	67.3 (11.9)	67.1 (12.0)	NS
BMI (kg/m^2^)	24.2 (3.3)	23.6 (3.3)	NS
ECOG PS, *n* (%)			
0–1	94 (93.1%)	201 (95.7%)	NS
≥2	7 (6.9%)	9 (4.3%)	
L3 Area (cm^2^)			
SAT	133.3 (71.2)	109.9 (59.2)	0.002
VAT	122.8 (85.8)	95.6 (61.2)	0.001
SM	133.6 (32.7)	138.2 (28.6)	NS
L3 Index (cm^2^/m^2^)			
SATI	49.0 (28.3)	39.4 (22.4)	0.001
VATI	44.1 (29.1)	33.6 (21.1)	<0.001
SMI	47.7 (9.1)	48.6 (8.0)	NS
No. of toxicities	3.5 (1.6)	1.7 (1.6)	<0.001

Data are presented as means (standard deviations) for continuous variables and as percentages for categorical variables. *p*-values are from the chi-square test or Fisher’s exact test when categorical and Student’s *t*-test when continuous. BMI: body mass index; DLT: dose-limiting toxicity; NS: not significant; SAT: subcutaneous adipose tissue; SATI: subcutaneous adipose tissue index; SM: skeletal muscle; SMI: skeletal muscle index; VAT: visceral adipose tissue; VATI: visceral adipose tissue index.

**Table 3:** Comparison of the sunitinib toxicities between patients with a high/low visceral adipose tissue index.

	Low Visceral Adipose Tissue Index	High Visceral Adipose Tissue Index	*p*
	(*n* = 155)	(*n* = 156)	
Early DLT, *n* (%)			
Present	37 (23.9%)	64 (41.0%)	0.001
Absent	118 (76.1%)	92 (59.0%)	
No. of toxicities, mean (SD)	1.5 (1.3)	3.1 (1.9)	<0.001

Data are presented as means (standard deviations) for continuous variables and as percentages for categorical variables. *p*-values are from the chi-square test or Fisher’s exact test when categorical and Student’s *t*-test when continuous. DLT: dose-limiting toxicity.
